# Trapezius muscle activity increases during near work activity regardless of accommodation/vergence demand level

**DOI:** 10.1007/s00421-015-3125-9

**Published:** 2015-02-20

**Authors:** H. O. Richter, C. Zetterberg, M. Forsman

**Affiliations:** 1 Department of Occupational and Public Health Sciences, Faculty of Health and Occupational Studies, Centre for Musculoskeletal Research, University of Gävle, 801 76 Gävle, Sweden; 2 Section of Occupational and Environmental Medicine, Department of Medical Sciences, Uppsala University, Uppsala, Sweden; 3 Institute of Environmental Medicine, Karolinska Institutet, Stockholm, Sweden

**Keywords:** Attention fatigue, Accommodation, Békésy test, Compensatory effort, Contrast threshold tracking, Electromyography, Visual ergonomics

## Abstract

**Aim:**

To investigate if trapezius muscle activity increases over time during visually demanding near work.

**Methods:**

The vision task consisted of sustained focusing on a contrast-varying black and white Gabor grating. Sixty-six participants with a median age of 38 (range 19–47) fixated the grating from a distance of 65 cm (1.5 D) during four counterbalanced 7-min periods: binocularly through −3.5 D lenses, and monocularly through −3.5 D, 0 D and +3.5 D. Accommodation, heart rate variability and trapezius muscle activity were recorded in parallel.

**Results:**

General estimating equation analyses showed that trapezius muscle activity increased significantly over time in all four lens conditions. A concurrent effect of accommodation response on trapezius muscle activity was observed with the minus lenses irrespective of whether incongruence between accommodation and convergence was present or not.

**Conclusions:**

Trapezius muscle activity increased significantly over time during the near work task. The increase in muscle activity over time may be caused by an increased need of mental effort and visual attention to maintain performance during the visual tasks to counteract mental fatigue.

## Introduction

Accommodation refers to the lenticular-based change made in the refractive state of the eye to attain and maintain a maximally high contrast foveal retinal image. There are four components that contribute to the overall accommodative response, namely, blur, disparity, and proximal and tonic accommodation (Ciuffreda 1998). The accommodation/vergence response consists of a tightly coupled triad of eye movements: dioptric (D) adjustment of the crystalline eye lens, convergence/divergence of both eyes toward the locus of fixation, and pupillary constriction/dilatation. The ciliary muscle adjusts the curvature of the lens, thereby changing its refractive power, allowing the formation of clear retinal images of objects located at a different distances (Ciuffreda 1998; Franzén et al. 2000).

The accommodative response degrades rapidly if the stimulus target (e.g., an alphanumerical character) is not projected directly into the fovea, the central portion of the retina where sensory photoreceptors responsible for high-resolution processing are located (Campbell 1954; Gu and Legge 1987). Campbell (1954) concluded that the photoreceptors involved in the accommodation reflex are the foveal cones and that in the absence of a foveal stimulus the accommodative reflex is not fully elicited. To keep the stimulus target projected into fovea, there is a need for compensatory eye-movements and eye-neck (head) stabilization. A neural command ought to impact on neck/shoulder muscle function through increased static muscle activity. Comparatively little is known about this sort of stabilizing eye-neck muscle synergy (Pelz et al. 2001; Richter et al. 2010).

Neck/shoulder muscle activation, measured with surface electromyography (EMG), during simulated near work, has shown that large accommodation response, when the ciliary muscle is highly contracted, is coupled to an increase in trapezius muscle activity level (Richter et al. 2010, 2011). A recent study with relatively low demands on accommodation and convergence, comparable to visual demands needed when working with, e.g., a smart phone or a tablet, showed that incongruence between accommodation and convergence may be an important factor in the relation between demanding near work and trapezius muscle activity (Zetterberg et al. 2013).

A sustained accommodation response, as an indirect measure of ciliary muscle load, may be hypothetically assumed to represent an individual characteristic which, if high in a relative sense due to non-optimal visual ergonomic work conditions (Bababekova et al. 2011; Rosenfield et al. 2012), over time could cause a progressive increase in trapezius muscle activation. In addition, progressively more attentional effort to execute a given level of accommodation response (Prsa et al. 2010; Vilupuru et al. 2005) may be expected as times goes by, due to depleted attention resources (Hockey 1997). Although increases in trapezius activity due to attentional demands at low-level static exertions have been reported (Iwanaga et al. 2000; Mehta and Agnew 2012), the circumstances under and the manner in which trapezius muscle activity results from sustained visual demands are still unclear in many fundamental ways. To date, in previous studies on the relation between accommodation response and trapezius muscle activity during demanding near work, the dimension of time has not been included (Lie and Watten 1987; Richter et al. 2010; Richter and Forsman 2011; Zetterberg et al. 2013).

Against the background above, the purpose of the current study was to apply the dimension of time into data analyses of a previously published study (Zetterberg et al. 2013). Because many near vision tasks contain a combination of high sustained accommodation demands and also continue for a prolonged time, these are highly relevant research questions with public health ramifications. The aim was to investigate if trapezius muscle activity increases over time during visually demanding near work.

## Methods

### Participants

Sixty-six participants (median age 38, range 19–47, 54 females and 12 males) were recruited, 33 with neck pain and 33 healthy controls. To exclude participants with eye diseases, the participants were examined by a licensed optometrist. All participants had normal corrected visual acuity (decimal ≥1.0). All participants were recruited through advertisement. Informed consent was obtained from each participant. The study was approved by the Uppsala University Medical Ethical Review Board, Uppsala, Sweden (2006:027).

### Procedure and preparations

Participants visited the laboratory on one occasion and undertook a visually demanding task, the Békésy contrast threshold tracking task (Richter and Knez 2007). The vision task lasted for 7 min, and was performed four times: each time with different trial lenses mounted on trial frames. Each vision task was preceded by a baseline (rest) when the participant sat relaxed with eyes closed for 3 min. The session started with preparations where refractive errors were measured using an auto refractor (Power Refractor R03, Plusoptix, Nürnberg, Germany) (Blade and Candy 2006). Then, trial lenses were selected with respect to any spherical refractive errors detected, i.e., the power of the refractive error was added to the trial lens for the right and the left eye (in steps of 0.25 diopters, D). The refractive error measurements conducted by the licensed optometrist’s generally agreed well with those reported by the auto refractor. The participant’s dominant eye was determined using a modified version of Dolmans method. The participant was set-up with surface electrodes for electrocardiography (ECG) laterally on each sixth rib, and with electromyography (EMG) bilaterally on the descending part of the trapezius muscle. EMG and ECG were continuously measured during the rests and vision tasks. Accommodation response was continuously measured with the auto refractor during vision tasks. For the auto refractor to detect the eyes and sample data, the eyes had to be aligned to the measurement axis of the auto refractor. To ensure a sufficient number of data points from the auto refractor, movements were prevented by supporting the participant’s head and trunk, and the participants were instructed to keep the posture and minimize movements.

### The vision task

The vision task consisted of 7 min of sustained foveal focusing on a fixation cross on a contrast-varying Gabor grating displayed on a computer screen (Sony F520 CRT monitor and a VSG video board, Cambridge Research System Ltd., Rochester, UK). Distance to screen was 0.65 m (1.5 D). The center of the grating was placed in the midline of the eyes, with the gaze angle approximately 15° downwards. For optimal stimulation of accommodation, the spatial frequency of the grating was set to 5 c/deg (Owens 1980). In addition, a standardized task instruction emphasized active accommodation: look at the fixation cross and the black-and-white grating. Carefully focus on the fixation cross so that it is maximally sharp and clear at all times (Atchison et al. 1994; Richter and Knez 2007; Zetterberg et al. 2013). Before the vision task started, the contrast of the grating was zero and only the fixation cross was visible. To start the vision task, the participant pushed a hand-held, low-force button, and the contrast of the grating started to increase (speed 0.8 percent/s). When the participant perceived the grating, he or she pushed the button and the contrast froze for a short period. After a pause of random length (1.5–3.5 s), the contrast of the grating decreased. When the grating became invisible to the participant, he or she pushed the button again. This procedure was repeated for 7 min. For a detailed image of the actual stimulus grating used in the experiment, see Zetterberg et al. (Fig. 2a–c, 2013)

### Viewing conditions and trial lenses

During the four vision tasks, different amounts of defocus blur were introduced by trial lenses mounted on trial frames (Oculus Inc., Dutenhofen, Germany). Lens order was randomized among participants using a Latin square. One of the four viewing conditions was binocular: −3.5 D (binocular minus, BM). The three other trial lens conditions were monocular: −3.5 D (monocular minus, MM): 0 D (monocular neutral, MN); +3.5 (monocular plus, MP). During the monocular lens conditions, the non-dominant eye was covered. The binocular condition was used to investigate the effect of incongruence between accommodation and convergence. The incongruent accommodation/vergence stimulus condition BM required the participants to maintain maximal focus on the target when the −3.5 D lens interrupted their line of sight while maintaining convergent visual axes on the same target. The monocular viewing conditions were used to isolate the effect of accommodation (i.e., ciliary muscle activity) and to investigate whether eye-lens accommodation is a mediating mechanism behind increased trapezius muscle activity (cf. Fincham and Walton 1957). The neutral viewing condition was used as a monocular reference. See Zetterberg et al. (2013).

**Fig. 1:**
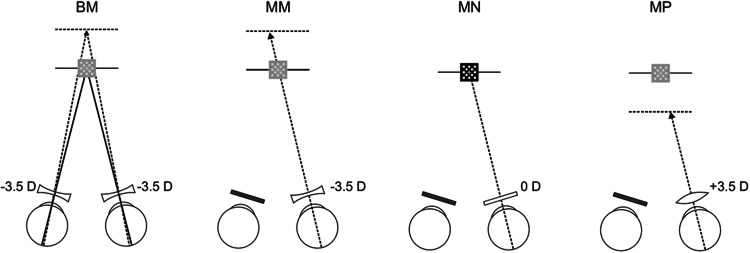
Schematic illustration of the accommodative and convergence stimulus (dioptric consequences) for the different lenses used in the experiment (*dotted line* = accommodative stimulus diopters; *full line* = convergence stimulus diopters). *BM* binocular −3.5 D, *MM* monocular −3.5 D, *MN* monocular 0 D, and *MP* monocular +3.5 D

The accommodation stimulus in each of the four viewing conditions was fixed and determined by the sum of the spherical power of the trial lens(es) and the distance to the screen (expressed in D). Accommodation stimuli were +5.0 D in the minus lens conditions (BM and MM), +1.5 D in the neutral-lens condition (MN) and −2.0 D in the plus lens condition (MP). The accommodation stimulus/response relationships in the neutral lens condition (MN) were expected to correspond closely to one another. The negative lens was used to facilitate increased accommodation, and the positive lens to facilitate decreased accommodation. Successful accommodation response performance in condition BM and MM required sustained contraction of the ciliary muscle to overcome the minus dioptric blur. In condition MP, the ciliary muscle should be relaxed to minimize the plus lens dioptric blur. The amount of stimulus blur presented in the plus lens condition is above the threshold for automatic error correction (i.e., out of range) and defocus blur is, therefore, expected to result (Fig. 1).

### Eye lens accommodation and contrast threshold

During the vision tasks, the auto refractor sampled data on pupil size and accommodation with a frequency of 25 Hz. The photorefraction technique analyzes the vergence of rays that are returned from the eye after reflecting an illuminated spot on the retina. In brief, the slopes of the brightening distribution in the pupil are converted to a refractive error. The possible range of corrected accommodative response measurements extends from about 0 D to +9 D, depending on pupil size in both eyes at the same time, and the direction of the pupil axes (Blade and Candy 2006). The auto refractor operates with a minimum pupil of 2.8 mm and tolerates eye movement’s ±25° from a central fixation. According to the manufacturer, the precision of measurements is ±0.25 D.

The auto refractor used in the current study (Power Refractor R03, Plusoptix, Nürnberg, Germany) was calibrated by comparing its measurements to a Topcon KR-8100 auto refractor (Topcon Medical System, Inc.). The ability of both auto refractors to detect refractive errors during unaided fixation on a designated target was found to be in excellent agreement with one another (See Richter et al. 2010). The corrected accommodation response values obtained during unaided fixation, when plus and minus lenses were placed in front of the eye, when both eyes were converging on the target at 1 m distance, were also examined. The corrected accommodation response values were found to be in excellent agreement with the net stimulus optical power at the eye. The auto refractor has been validated in the scientific literature (cf. Blade and Candy 2006).

The data from the auto refractor were processed in MATLAB 7.1 (MathWorks Inc., Natick, MA, USA). In each lens condition, participants with ≤25 % of sampled data from the auto refractor and accommodation response outside of the linear range were excluded from the analyses (Richter et al. 2010).

The majority of the data points were clustered together within a physiologically plausible range. A few cases with “to high” accommodation responses appeared to have been stimulated by the reflecting mirror, placed between the eyes and the auto refractor, at a distance closer to the eyes than the stimulus patterns (see Fig. 1 in Zetterberg et al. 2013). In two cases, the accommodation response appeared to have been placed at or near infinity. In these cases, the resulting response level may have been influenced by eye strain or fatigue. All outliers were deleted from analysis (number of excluded cases in BM = 28, MM = 23, MN = 14 and MP = 13). Only data from the dominant eye were used. See Richter et al. (2010).

To render the recorded refraction data into a format which allows comparison with the stimulus diopters, all instrument readings were corrected for by 1.0 D (in order to shift the default distance of 1 m to optical infinity). Thereafter, the effect of the externally added lens’ powers was corrected for see (Richter et al. 2010).

Mean of the accommodation response during the 1st through the 7th minute of the vision task was computed to assess the stability over time (accommodation response_1-min averages_). A mean of the 7-min period was computed (accommodation response_7-min average_) for the statistical analyses.

The contrast of the Gabor grating displayed on the screen varied throughout the task [Contrast = (*L*_max _− *L*_min_)/(*L*_max_ + *L*_min_), *L* = luminance], and the contrast threshold was determined using the Békésy tracking method (Richter and Knez 2007). Individual contrast threshold was recorded throughout the vision task each time the participant indicated with the low-force button when he/she perceived the grating and when the grating became invisible to the participant.

To assess the stability over time, average values of the contrast threshold of the grating during the 1st through the 7th minute of the vision task were computed (contrast threshold_1-min averages_). A mean of the 7-min period was also computed (contrast threshold_7-min average_) to make it possible to assess the relationship with the accommodation response.

### Electromyography and electrocardiography

EMG and ECG were recorded both during rest periods and during vision tasks. EMG and ECG signals were amplified, band-pass filtered (EMG: 10-500 Hz, ECG; 0.05–35 Hz), and sampled at 2,000 Hz (EMG100C, BIOPAC Systems, Inc., Santa Barbara, CA, USA). The EMG and ECG data were processed in MATLAB 7.1. ECG was used to reduce disturbances from heart signals on raw EMG (Widrow et al. 1975; Woolfson et al. 1990; Zetterberg et al. 2013). ECG was also used to assess the heart rate variability (HRV), a marker of autonomic reactivity (e.g., due to arousal) during the experiments. Analyses of the variation of intervals between consecutive heartbeats have been shown to quantify the autonomic heart regulation and the balance between sympathetic and parasympathetic activation. Because of the very short periods (consecutive 1-min periods) as the basis for HRV calculation, the standard deviation of the times between the R peaks was chosen to quantify HRV. Mean of the HRV during the 1st through the 7th minute was computed to assess the stability over time (HRV_1-min averages_). A mean of the 7-min period was computed (HRV_7-min average_) for the statistical analyses.

The EMG recordings were root-mean-square (RMS) converted in 0.1-s periods, adjusted for noise, normalized to submaximal reference contractions, and expressed in % RVE (reference voluntary electrical activity) (Mathiassen et al. 1995). The 50th percentile, i.e., the median, of the normalized RMS values was used to quantify muscular activity level (Jonsson 1982; Richter et al. 2010; Thorn et al. 2007). The median of the RMS of the 3-min rest period (EMG_rest 3-min average_) was used as an estimate of the rest level in the main analyses. In addition, median RMS values of the 1st, 2nd, and 3rd minute were used to assess the stability over time (EMG_rest 1-min averages_).

To analyze whether muscle activity increased over time during the vision task, median values of the 1st through the 7th minute were computed (EMG_fix 1-min averages_). All statistical tests on EMG were run on a mean of the left and the right trapezius muscle (Zetterberg et al. 2013). A logarithmic transform was applied on all EMG variables prior to the statistical analysis to correct for a tilt in the distribution of data, which was skewed, with most participants exhibiting very low or low activation levels with a tail in the right direction.

### Statistical testing

Statistical analyses were performed using PASW 20.0 for Windows (SPSS Inc., Chicago, IL, USA) and the significance level was *α* = 0.05. All variables were first tested for normality using the Kolmogorov–Smirnov test. The choice of statistical tests was then based on the data distribution.

General estimating equation (GEE) was used to analyze if trapezius muscle activity increases over time during visually demanding near work. The choice of this statistical method was motivated by the fact that an autocorrelation can be assumed in time series, i.e., neighboring values are likely to be correlated. Thus, GEE provides a general framework for analysis of time series data and does also relax other key assumptions of traditional regression models (Ballinger 2004; Fitzmaurice et al. 2011; Ghisletta and Spini 2004; Hanley et al. 2003).

GEE was used to assess the relationship between 1 min means of contrast thresholds (contrast threshold_1-min averages_) and accommodation responses (accommodation response_1-min averages_) with time (min 1–7) to ascertain if the two sets of response measures were stable. Spearman correlation coefficients were in addition computed between mean accommodation response (accommodation response_7-min average_) and mean contrast thresholds (contrast threshold_7-min average_) for each viewing condition for the purpose of assessing the internal validity of the results obtained with two different measurement techniques.

Before the main analysis began, four control GEE analyses were run, one for each rest period immediately preceding the visual tasks: independent variables were logEMG_rest 1-min averages_ and time (min 1–3). The purpose was to ascertain that the trapezius activity did not increase over time during the rest periods.

To assess changes in heart rate variability over time during the vision tasks, as a marker of autonomic reactivity, GEE was computed between HRV_fix 1-min averages_ and time (min 1–7) for each viewing condition.

To investigate if the trapezius muscle activity increases over time during visually demanding near work, four GEE analyses were run, one for each lens condition. The GEE explores the main effect of time (min 1–7) on the dependent variable trapezius muscle activity (logEMG_fix 1-min averages_). To control for the effect of group (neck or control group), trapezius muscle activity during rest, heart rate variability, and accommodation response, the variables group, logEMG_rest 3-min average_, HRV_fix 1-min averages,_ and accommodation response _7-min average_ were included as covariates in the GEE model. If accommodation response_1-min averages_ was not stable over time, it was used in the GEE model. Otherwise, it was replaced by accommodation response_7-min average_. In the initial analytic step, the GEE was used to test the contributions of all independent variables. In the subsequent analytic step, if one or more variable did not make a significant contribution to the model (*p* ≥ 0.10), this/these variable(s) was removed, and the model was re-estimated.

## Results

### Accommodation responses and contrast thresholds

**Fig. 2:**
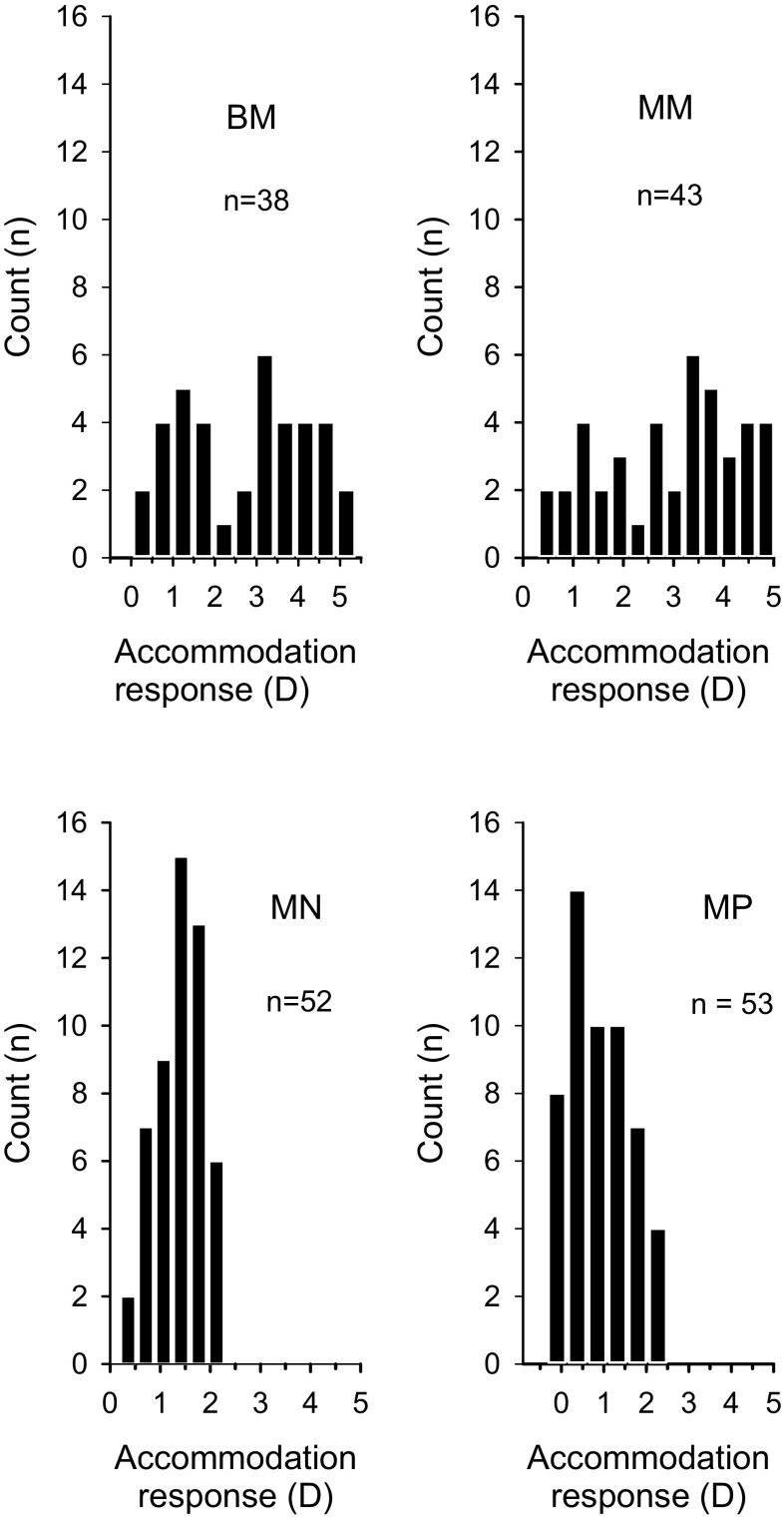
Frequency counts of individual mean accommodation response for the 7-min vision task for the four lens conditions (accommodation response_7-min average_). *BM* binocular −3.5 D, *MM* monocular −3.5 D, *MN* monocular 0 D, and *MP* monocular +3.5 D

**Fig. 3:**
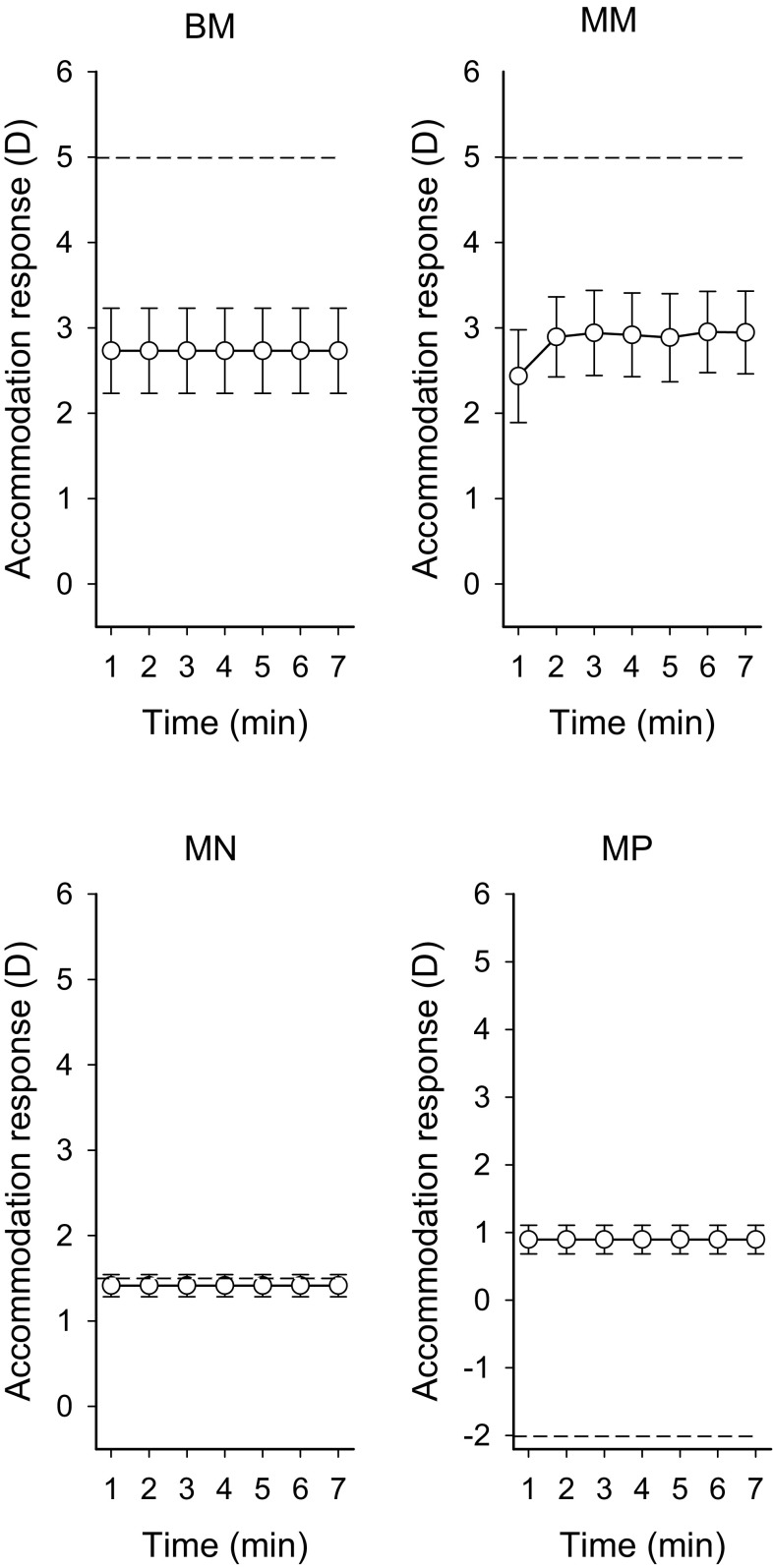
Mean accommodation response for minutes 1 through 7 for the vision task in the four lens conditions (accommodation response_1-min averages_). *Horizontal* *stippled lines* denote stimulus diopters. *Error* *bars* represent 95 % CI. *BM* binocular −3.5 D, *MM* monocular −3.5 D, *MN* monocular 0 D, and *MP* monocular +3.5 D

In Table 1, the median, minimum and maximum accommodation response values over the 7-min vision task for the different lens conditions are displayed. Figure 2 shows frequency counts of the individual mean accommodation response values over the four different lens conditions. The individual accommodation response values correlated with age in BM (*r*_s_ −0.58, *p* < 0.0001) and MM (*r*_s_ −0.554, *p* < 0.0001) but not MN and MP (not shown). The mean accommodation response computed on individual 1-min means (accommodation response_1-min averages_) is visualized in Fig. 3.

**Table 1:** Median values of accommodation response over the 7-min vision tasks for the four lens conditions (accommodation response_7-min average_): min–max values in brackets

Viewing condition	Total	*N*	Control group	*N*	Neck group	*N*
BM	3.06 (0.38–4.25)	38	3.15 (0.38–5.25)	21	3.00 (0.47–4.59)	17
MM	3.23 (0.29–5.09)	43	3.23 (0.29–5.09)	24	3.21 (0.66–4.78)	19
MN	1.50 (0.21–2.29)	52	1.44 (0.21–2.29)	30	1.51 (0.51–2.15)	22
MP	0.84 (−0.64–2.51)	53	0.85 (0–1.67)	24	0.78 (−0.34–2.51)	29

*BM* binocular minus, *MM* monocular minus, *MN* monocular neutral, *MP* monocular plus, *N* number of valid cases

**Fig. 4:**
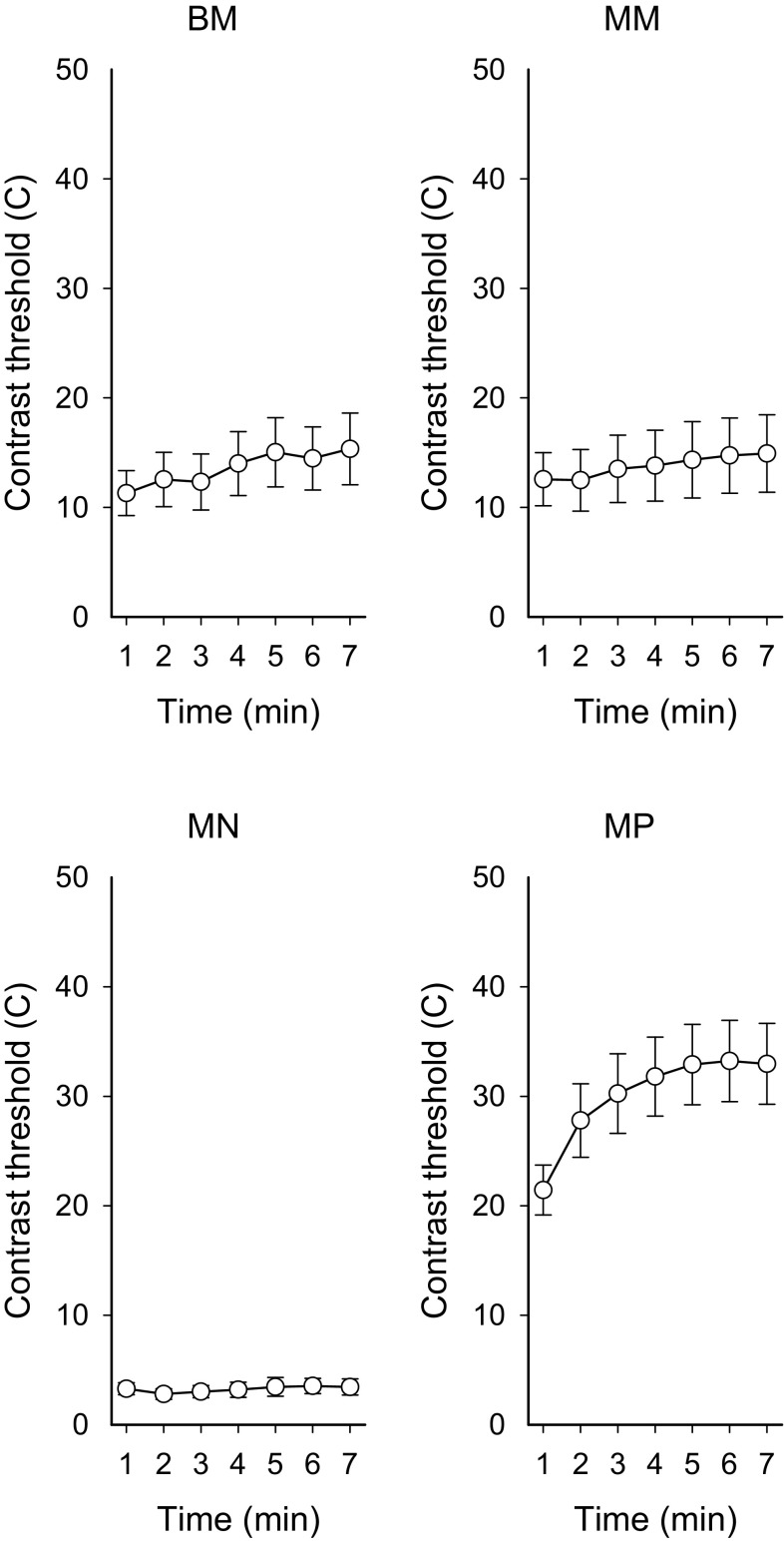
Mean of contrast thresholds for minutes 1 through 7 for the vision task for the four lens conditions (contrast thresholds_1-min averages_). *Error bars* represent 95 % CI. *BM* binocular −3.5 D, *MM* monocular −3.5 D, *MN* monocular 0 D, and *MP* monocular +3.5 D

The GEE analyses, time versus accommodation response_1-min averages_, showed no significant temporal relationship in BM (*β*-coefficient = −0.04, *p* = 0.094), MN (*β*-coefficient = −0.015, *p* = 0.076) or MP (*β*-coefficient = −0.004, *p* = 0.79). In MM, a significant increase was detected (*β*-coefficient = 0.084, *p* < 0.001) (see Fig. 3 min 1–2). Figure 4 visualises the mean of individual contrast threshold values min 1–7. The GEE analyses, time versus contrast threshold_1-min averages_, showed a significant positive relationship between time and contrast threshold in lens condition BM (*β*-coefficient = 0.568, *p* < 0.001), MN (*β*-coefficient = 1.38, *p* = 0.022) and MP (*β*-coefficient = 1.308, *p* < 0.0001). In MM, the relationship was non-significant (*β*-coefficient = 0.209, *p* = 0.237). Contrast thresholds_7-min average_ in the minus lens conditions were significantly correlated with the accommodation response_7-min average_ (BM *r*_s_ −0.686, *p* < 0.0001, *n* = 38: MM *r*_s_ −0.728, *p* < 0.0001, *n* = 43). In the neutral and positive lens conditions, the relationship was non-significant (MN *r*_s_ −0.101, *p* = 0.055, *n* = 52; MP *r*_s_ 0.060, *p* = 0.264, *n* = 53) (not shown).

### Trapezius muscle activity during rest

**Fig. 5:**
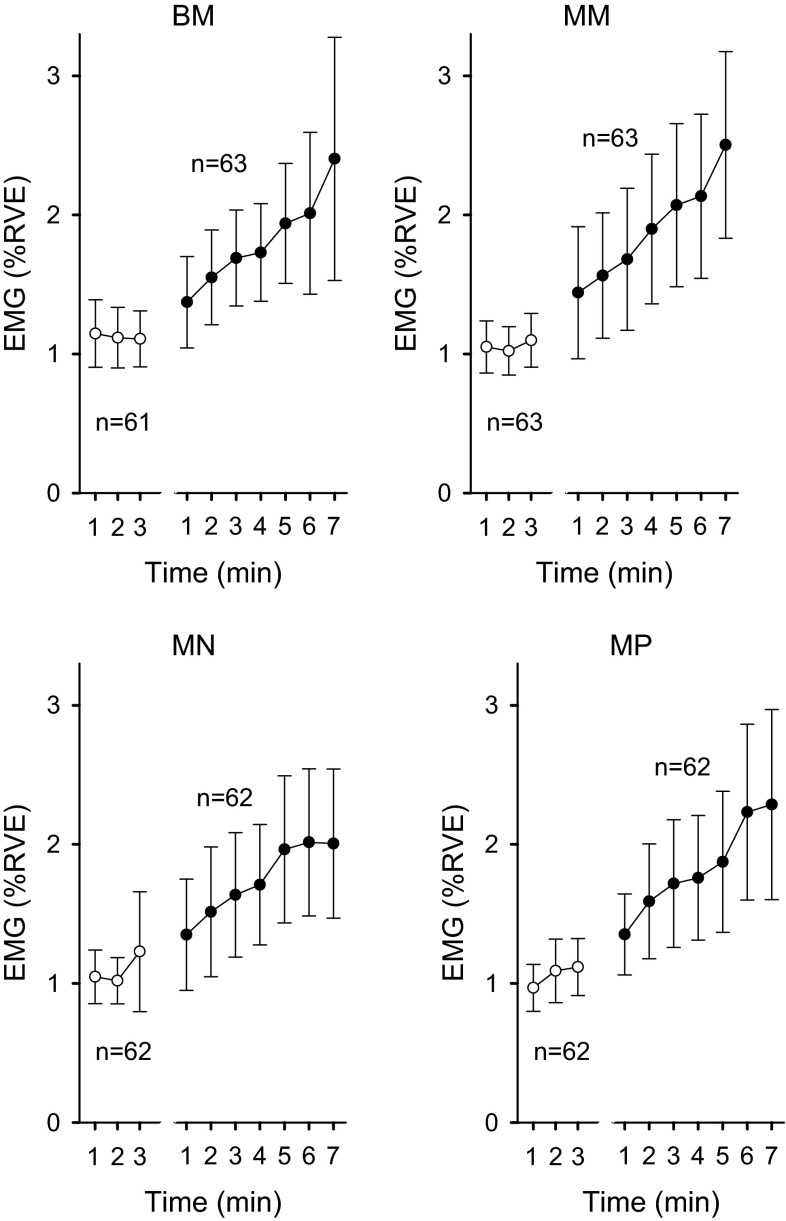
Mean trapezius muscle activity (% RVE) during rest (*white circles*) and during the 7-min vision task (*black circles*) as a function of time for the four lens conditions. *Error bars* represent 95 % CI. *BM* binocular −3.5 D, *MM* monocular −3.5 D, *MN* monocular 0 D, and *MP* monocular +3.5 D

The white circles in Fig. 5 show the mean trapezius muscle activity (% RVE) during rest (EMG_rest 1-min averages_). The four control GEE analyses on trapezius muscle activity during rest did not reveal any significant change in muscle activity over time in the four rest periods preceding the vision task. The *β*-coefficient was 0.000 (*p* = 0.980) in condition BM, 0.009 (*p* = 0.470) in MM, 0.010 (*p* = 0.451) in MN, and 0.024 (*p* = 0.075) in MP.

### Heart rate variability during visual task

The four GEE control analysis on heart rate variability showed that HRV_fix 1-min averages_ decreased over time in condition BM (*β*-coefficient = −0.740, *p* = 0.024). No other changes in heart rate variability over time were detected (MM *β*-coefficient = −0.248, *p* = 0.387; MN *β*-coefficient = −0.138, *p* = 0.586 and MP *β*-coefficient = −0.288, *p* = 417).

### Trapezius muscle activity during visual task

**Fig. 6:**
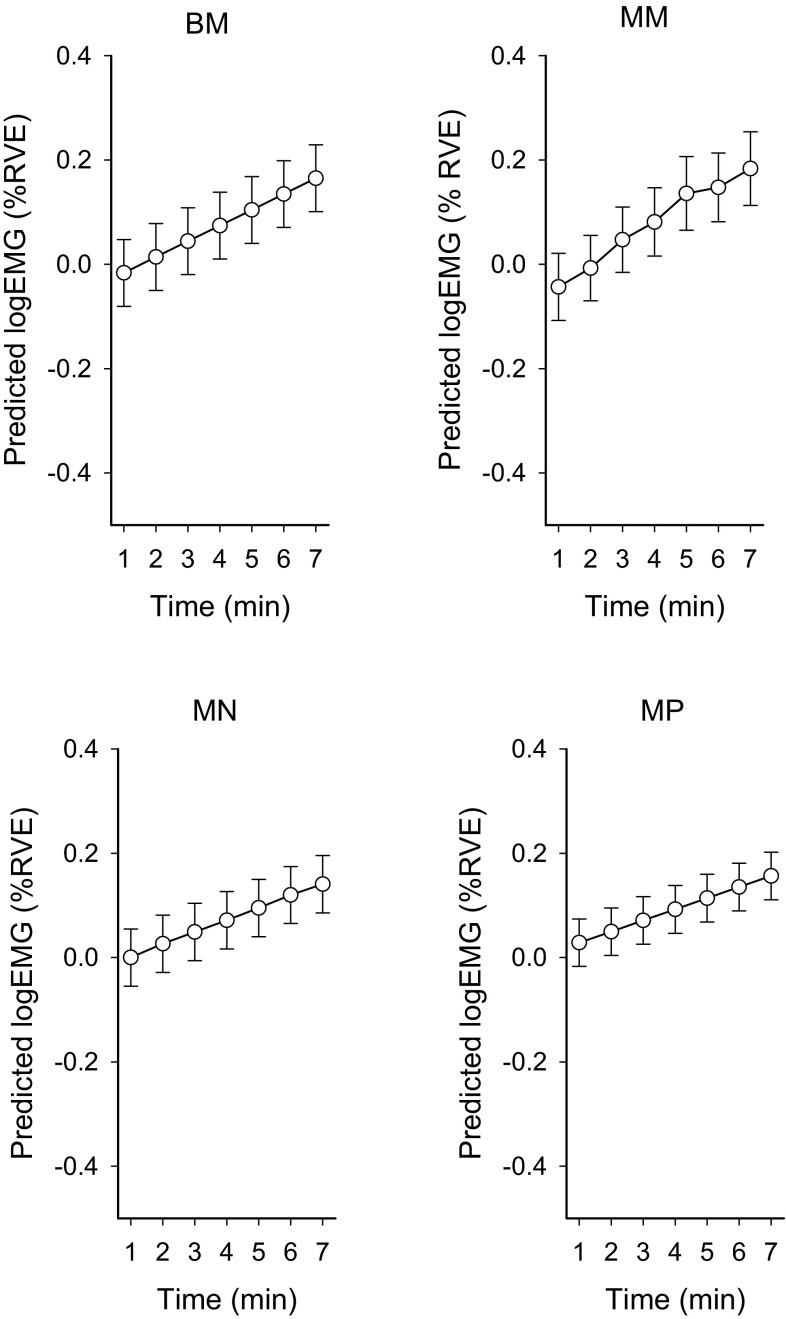
Predicted mean values from the GEE models (Table 2) for the trapezius muscle activity during the 7-min vision task. *Error bars* represent 95 % CI. *BM* binocular −3.5 D. *MM* monocular −3.5 D, *MN* monocular 0 D, and *MP* monocular +3.5 D

The black circles in Fig. 5 show the group means trapezius muscle activity (% RVE) during the visual tasks (EMG_fix 1-min averages_). The results from the four GEE analyses are presented in Table 2. These four GEE analyses revealed that trapezius muscle activity increased significantly over time in the vision task in both the incongruent binocular condition (BM), and the congruent monocular conditions (MM, MN and MP) (*p* < 0.003). There was no main effect of group or trend thereof in any of the active accommodation conditions (BM, MM) or the other conditions (MN, MP). Introducing the term group in the statistical model also did not impact on the effect of accommodation response (i.e., the previous results remain the same). The effect of accommodation response on trapezius muscle activity was significant in the two minus lens conditions with high accommodative demands, irrespective of whether incongruence was present or not (BM *p* = 0.007 and MM *p* = 0.048), but there was no significant effect of accommodation response with the less demanding lenses (MN and MP). Since there was a significant effect of time on accommodation response in condition MM (see Sect. “Accommodation responses and contrast thresholds”; Fig. 3), the variable accommodation response_1-min averages_ was used when analyzing condition MM. GEE analyses also revealed that trapezius muscle activity during the rest period (EMG_rest 3-min average_) accounted for a significant portion of muscle activity in the vision task in all four lens conditions (*p* < 0.001). Figure 6 shows the predicted mean values from the independent effects’ models (BM, MM, MN, and MP).

**Table 2:** Summary of the main effects from the GEE analyses analysing if trapezius muscle activity increases over time during visually demanding near work

Viewing condition	Independent variable	Slope (*β*)	95 % CI		*p* value	QIC*
			Lower	Upper		
BM	Intercept	−0.150	−0.300	4.4E−5	0.050	51.2
	LogEMG_rest full 3 min_	0.579	0.271	0.887	<0.001	
	Time	0.030	0.012	0.049	<0.001	
	AR_full 7 min_	0.065	0.018	0.111	0.007	
MM	Intercept	−0.093	−0.206	0.019	0.104	37.2
	LogEMG_rest full 3 min_	0.615	0.410	0.819	<0.001	
	Time	0.038	0.018	0.059	<0.001	
	AR_min 1–7_	0.035	0.000	0.070	0.048	
MN	Intercept	0.096	−0.036	0.227	0.153	59.5
	LogEMG_rest full 3 min_	0.733	0.408	1.057	0.001	
	Time	0.023	0.008	0.039	0.003	
	HRV_min 1–7_	−0.001	−0.001	2.2E−5	0.043	
MP	Intercept	0.075	−0.008	0.159	0.075	63.8
	LogEMG_rest full 3 min_	0.620	0.297	0.944	<0.001	
	Time	0.021	0.007	0.036	0.003	

*BM* binocular −3.5 D, *MM* monocular −3.5 D, *MN* monocular 0 D, and *MP* monocular +3.5 D. *AR* accommodation response. * Quasi-likelihood under independence model criterion: an information criterion to be used to help choose between correlation structures, to be interpreted as the smaller the better (Wei 2001)

The dependent variable was logEMG_fix 1-min averages_

## Discussion

The present findings showed a significant increase in trapezius muscle activity over time during the visually demanding near work, in all four viewing conditions, see Table 2 and Figs. 5 and 6. In the two viewing conditions with high accommodative demands, the accommodation responses were positively associated with the muscle activity levels, see Table 2.

### Oculomotor performance during visual task

In the neutral condition (MN), accommodation response corresponded closely to the stimulus dioptric distance to the target. This outcome was expected because the neutral condition was deliberatively designed to be easy to comply with. In the active accommodation conditions, the majority of the participants nullified the added minus dioptric blur to varying degrees. Beginning presbyopia was associated with accommodation response values <1.50 D in a subset of participants >38 years of age. For the plus lens condition (MP), since there was effectively no stimulus to accommodation, the accommodative system was rendered open loop and moved towards the tonic (or dark focus) accommodation level. The current median accommodation response of 0.84 D is close to the nominal mean tonic accommodation level (Ciuffreda 1998).

The control GEE analyses which related accommodation response and contrast thresholds to time indicated that participants generally maintained their level of accommodation response during the visual tasks but that the contrast threshold decreased. The general drop off in contrast threshold, in all conditions except MM, suggests that the visual demands were too high and that the participants were unable to fully compensate for the mental fatigue induced by high visual attention.

### Effect of time on trapezius muscle activity

Trapezius muscle activity increased over time in all four viewing conditions regardless of level of accommodative demand. This indicates that the variation in accommodation/vergence responses was not associated with the increase in muscle activity found in this study. However, the main effects of time and accommodation response were significant in the minus lens conditions (BM and MM) and these two variables (time and accommodation response), therefore, contribute to an increased trapezius muscle activity in an additive way. The overlap of the 95 % confidence intervals for the slope estimates in Table 1 indicates that the level of trapezius muscle activity across viewing conditions did not differ between one another. This result agrees with the results reported by Zetterberg et al. (2013).

In our previous study (Zetterberg et al. 2013), the level of eye lens accommodation was significantly related to a small amount of trapezius muscle activity only in the incongruent binocular minus lens viewing condition. Thus, the incongruence between accommodation and convergence may be an important factor in the relation between visually demanding near work and trapezius muscle activity. In the present study, trapezius muscle activity was related to accommodation responses in both viewing conditions with minus lenses (BM and MM), irrespective of whether incongruence was present or not, even though the effect of accommodation was somewhat more pronounced during incongruent viewing (i.e., in BM) (cf. Table 2). This difference in results was presumably due to the fact that the GEE analysis of accommodative response in condition MM was based on minute by minute means unlike the analysis in Zetterberg et al. (2013) which was based on a 7-min mean.

During rest periods, there were no indications of the increase in muscle activity over time, and the increase in muscle activity during the visual task dropped back to rest level again shortly after the visual task. Even though the sitting posture and the sitting instructions were the same during both the rest periods and the visual tasks [participants were instructed to sit relaxed, with eyes closed (during rest), and to fixate on the screen (during the tasks)], it could be possible that the participants adopted a more relaxed posture during rest than during the visual tasks.

While skeletal muscle fatigue is defined as a progressive decline in maximum voluntary force produced by a muscle or a muscle group (Tanaka and Watanabe 2012), comparatively less is known about visual fatigue. It was recently suggested that the extraocular and ciliary muscles of the eye are, unlike skeletal muscles, resistant to fatigue (Prsa et al. 2010; Vilupuru et al. 2005). Hence, it is more likely that the trapezius muscle increases associated with the visually demanding tasks in this study were related to mental fatigue due to high visual attention, rather than eye muscle fatigue. To maintain performance when mental fatigue is present, the negative feedback system that normally protects the brain from overload could be suppressed. Nakagawa et al. (2013) recently studied brain activity with functional Magnetic Resonance Imaging during mental fatigue. The mental fatigue was induced by a visual and auditory attention demanding task. The main results showed inhibition of a midbrain structure which normally triggers rest to maintain a healthy balance between rest, and physical and mental load. Participants in the current study probably invested progressively more visual attention, which in turn likely led to mental fatigue and compensatory mental effort to maintain performance during the visual tasks (Grauer and Dunn 1980; Nakagawa et al. 2013). The trapezius muscle increases observed in the current study may, in analogy to the findings presented by Nakagawa et al. (2013), be caused by an increase in mental effort to compensate for the mental fatigue induced by increased visual attention (Faber et al. 2012; Nakagawa et al. 2013; Tanaka and Watanabe 2012; Wang et al. 2011), as an increase in mental effort may increase muscle activity (Iwanaga et al. 2000; Mehta and Agnew 2012).

### Deficient visual ergonomics a risk factor for trapezius muscle myalgia?

The results of this study underscore the significance of attention demanding near work tasks on the gradual buildup of muscle activation in the neck/scapular area, along with the effects from accommodation/vergence load. Long-term musculoskeletal consequences of accommodation/vergence overload in real working life have not yet been studied in any detail (Blehm et al. 2005; Iribarren et al. 2001; Rosenfield et al. 2012; Toomingas et al. 2014; Watten and Lie. 1992; Watten et al. 1994). Hence, whether the type of accommodation/vergence overload studied here under controlled laboratory conditions contributes to the progression of musculoskeletal disorders in the neck/shoulder area is unknown. The accommodation/vergence system does exhibit signs of overload in contemporary working life, including eye discomfort, transient myopia, and associated phoria (e.g., Blehm et al. 2005; Collier and Rosenfield 2011; Gobba et al. 1988; Jaschinski-Kruza 1984, 1988; Kreczy et al. 1999; Krupinski and Berbaum 2009; Ong and Ciuffreda 1995; Rosenfield et al. 2012; Watten and Lie 1992). Accommodation/vergence overload, caused by non-ergonomic near work, may also emerge as quickly as within one regular workday (Gobba et al. 1988; Jaschinski-Kruza 1984). Modest musculoskeletal symptoms may be a prognostic for the development of more severe symptoms (Nordlund and Ekberg 2004).

### Limitations and methodological aspects

The duration of exposure at the predefined levels of oculomotor load was restricted (7-min in each viewing condition) in the present study. Experiments in the laboratory are necessarily very short when compared to the extended exposure to deficient visual near work which may occur during professional computer work. Because the eyes had to be aligned to the axis of the auto refractor, movements from the neck/scapula area were not allowed. To more closely mimic real-life working conditions, it is important to, in future experiments, allow eye–neck interactions to develop naturally and over longer periods of time.

## Conclusion

Trapezius muscle activity increased significantly over time during the 7-min visually demanding near work task. In viewing conditions with relatively high accommodative demands, the level of accommodation response explained some part of the trapezius muscle activity, irrespective of whether incongruence between accommodation and convergence was present or not. The increase in muscle activity over time may be caused by an increased need of mental effort to maintain performance during the visual tasks to counteract mental fatigue due to high visual attention.

## Abbreviations

BM: Binocular minus −3.50 D blurring of grating and compensatory reduction of optical blur by binocular plus accommodation/vergence
D: Dioptre: the refractive power of a lens or an optical system. It is the reciprocal of distance in meters so that 1 dioptre corresponds to 1 m, 2 dioptres corresponds to 0.50 m, etc
ECG: Electrocardiography
EMG: Electromyography
GEE: General estimating equation
MM: Monocular minus −3.50 D blurring of grating and compensatory reduction of optical blur by monocular plus accommodation/vergence
MN: A no-blur monocular reference viewing condition
MP: Attempts at reduction of optical blur by monocular negative accommodation/vergence in response to +3.50 D blurring of grating
RMS: Root mean square
RVE: Reference voluntary electrical activity

## List of symbols

**Subscripts**
Fix: 7-Min sustained visual fixation period
Rest: 3-Min rest period
